# *Chryseobacterium indologenes* Septicemia in an Infant

**DOI:** 10.1155/2014/270521

**Published:** 2014-07-10

**Authors:** Turkan Aydin Teke, Fatma Nur Oz, Ozge Metin, Gulsum Iclal Bayhan, Zeynep Gökce Gayretli Aydin, Melek Oguz, Gonul Tanir

**Affiliations:** ^1^Dr. Sami Ulus Maternity and Children's Research and Training Hospital, Division of Pediatric Infectious Diseases, No. 44, Altındağ, 06080 Ankara, Turkey; ^2^Dr. Sami Ulus Maternity and Children's Research and Training Hospital, Department of Pediatrics, 06080 Ankara, Turkey

## Abstract

*Chryseobacterium indologenes* is a rare cause of infection in children. The organism causes infections mostly in hospitalised patients with severe underlying diseases. The choice of an effective drug for the treatment of infections due to *C. indologenes* is difficult as the organism has a limited spectrum of antimicrobial sensitivity. We present a case of nosocomial septicemia caused by *C. indologenes* in an infant with congenital heart disease who was successfully treated with trimethoprim sulfamethoxazole and also reviewed fourteen additional cases of *C. indologenes* infections reported in the English literature in this report.

## 1. Introduction

*Chryseobacterium indologenes* is nonmotile, catalase-positive, oxidase-positive, indole-positive, non-glucose-fermenting Gram-negative bacilli. It is not a component of human flora although it is widely distributed in nature.* C. indologenes* is a rare organism that has been reported to cause infections mostly in hospitalised patients with severe underlying diseases [1, 2]. The appropriate antibiotic choice is challenging as the organism shows resistance to multiple antibiotics. This report describes a case of* C. indologenes* nosocomial septicemia in an infant with congenital heart disease and reviews previously reported infections by* C. indologenes* in pediatric age group.

## 2. Case Report

**Figure 1:**
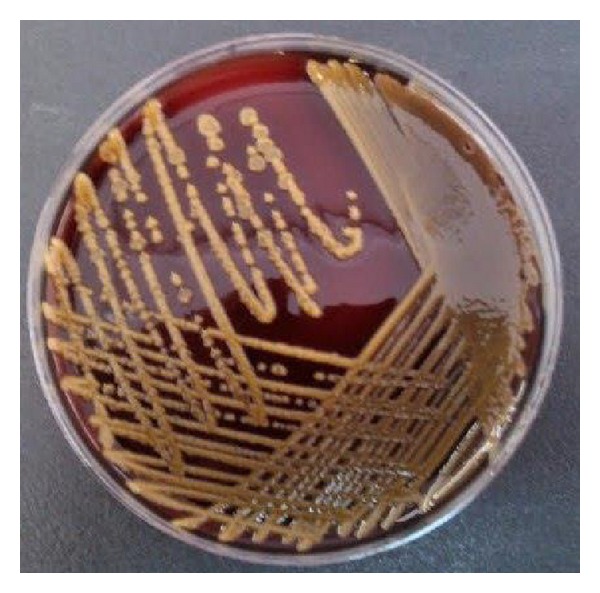
Yellow colonies of* Chryseobacterium indologenes* on blood agar.

A three-month-old female infant born at term by vaginal delivery presented to our hospital with cough and irritability. Atrioventricular septal defect was diagnosed when she was 15-day-old and she was on followup by cardiology clinic. On admission she had tachypnea and cardiac murmur on auscultation. All the other physical examinations of her were unremarkable. A complete blood count showed a white blood cell (WBC) count of 11.000/mm³ with 60% lymphocytes and C-reactive protein (CRP) level was 1 mg/L (range, 0–8 mg/L). Serum biochemical investigations were in normal range. Chest X-ray showed hyperinflation and peribronchiolar wall thickening. She was hospitalized for acute community acquired pneumonia and ampicillin sulbaktam plus clarithromycin was initiated. Respiratory virus multiplex polymerase chain reaction (PCR) obtained at admission revealed respiratory syncytial virus. Blood culture was performed since her clinical condition deteriorated and fever occurred on day 6.* Candida albicans* was isolated on blood culture and she was treated for nosocomial candidemia with caspofungin for 21 days, serial blood cultures were negative, and she was discharged in a good clinical condition. Four days after discharge, she was rehospitalized for the diagnosis of nosocomial pneumonia and empirically treated with meropenem and caspofungin intravenously. Blood and urine cultures showed no growth. During the hospitalization, fever up to 39°C occurred. She had a central venous catheter and her remaining physical examination was unremarkable. Laboratory investigations were as follows: hemoglobin 9.5 mg/dL, WBC 22.200/mm^3^ with 70% neutrophil, platelets 516.000/mm^3^, and CRP 21.1 mg/L. Chest X-ray showed no new infiltration and also abdominal ultrasonography and echocardiography did not show any infectious focus. Central venous catheter was removed because of obstruction. Specimen from peripheral blood culture yielded yellow colonies on blood agar (Figure 1).* C. indologenes* was identified by conventional methods and VITEK 2 ID-AST (bioMérieux, France) automatized system. Antimicrobial susceptibility testing of the organism revealed resistance to amikacin, gentamicin, ceftazidime, cefepime, piperacillin tazobactam, cefoperazone sulbactam, imipenem, meropenem, colistin, tetracycline, and ciprofloxacin and was susceptible to only trimethoprim sulfamethoxazole (TMP-SMX). TMP-SMX (20 mg/kg/day, intravenously) was initiated. Her fever resolved and blood culture became negative after 48 hours of the treatment. The antibiotic treatment was given for a course of 21 days. After discharge she was operated on in another hospital. She has been on followup in a good clinical condition for seven months.

## 3. Discussion

The genus* Chryseobacterium *(previously* Flavobacterium)* belongs to the family Flavobacteriaceae that are ubiquitous in nature and are inhabitants of soil and water and can be recovered from a variety of foods. They can be found in municipal water supplies despite adequate chlorination and can be recovered from the hospital environment [3]. Contamination of the medical devices containing water (respirators, intubation tubes, humidifiers, etc.) in hospital settings may lead to serious infections.* C. indologenes* colonies usually form a dark-yellow pigment in culture as a result of the production of the pigment flexirubin.

*C. indologenes* is a rare cause of human disease.* C. indologenes *was first isolated from a clinical specimen in 1993 from the tracheal aspirate of a patient with ventilator-associated pneumonia [4].* Chryseobacteria* represent only 0.27% (50 of 18,569) of the processed nonfermentative Gram-negative bacilli and 0.03% (50 of 155,811) of all bacterial isolates collected by the SENTRY Surveillance Program during the 5-year period 1997 to 2001 [5].* C. indologenes *infections have been shown as a cause for a variety of invasive infections especially in patients with risk factors, such as intravascular catheter-related bacteremia, bacteremia associated with malignancy and neutropenia, nosocomial pneumonia, cellulitis, meningitis, peritonitis, and surgical wound infections [2, 6–11]. A total of 14 cases of* C. indologenes* infections in pediatric age group were found in English literature. The diagnoses of the reported cases were bacteremia (six cases), meningitis (five cases), ventilator-associated pneumonia (two cases), and lumboperitoneal shunt infection (one case). Ten of 14 patients had medical devices, 12 of 14 patients had comorbidity, and 12 of 14 patients were ≤2 years old. The summary of cases is presented in Table 1.

*Chryseobacteria* have low pathogenicity and cause infections mostly in hospitalised patients with risk factors including underlying medical illness, age (newborn or elderly) underlying immunocompromising conditions, presence of indwelling intravascular devices, and prolonged exposure to broad-spectrum antibiotics. The present case had congenital heart disease, central venous catheter, and prolonged usage of broad-spectrum antibiotics as risk factors.

The choice of an effective drug for the empirical treatment of infections due to* C. indologenes *is difficult as the organism has a limited spectrum of antimicrobial sensitivity.* Chryseobacterium* organisms produce *β*-lactamases and are resistant to most *β*-lactam drugs, including the carbapenems and aztreonam [3]. In a study that included 215* C. indologenes *isolates, TMP-SMX and cefoperazone-sulbactam remained the most active agent especially for bloodstream infections. Authors concluded that, after introduction of colistin and tigecycline usage because of emerging resistant pathogens, the prevalence of* C. indologenes* infection increased [12]. According to the results of the SENTRY Antimicrobial Surveillance Program, the most active agents against* C. indologenes* are the newer quinolones (garenoxacin, gatifloxacin, and levofloxacin, ≥95% susceptibility) and TMP-SMX (95% susceptibility), followed by piperacillin-tazobactam (90% susceptibility). Ciprofloxacin, cefepime, ceftazidime, piperacillin, and rifampin showed reasonable activity (85% susceptibility) [5]. On the contrary, other *β*-lactams, aminoglycosides, chloramphenicol, linezolid, and glycopeptides are not appropriate for treating infections caused by this organism. According to more recent report it was suggested that only newer fluoroquinolones and TMP-SMX could possibly represent the most appropriate antimicrobial agents [1].

In conclusion* C. indologenes* may cause nosocomial septicemia in infants with the risk factors as underlying prolonged hospitalization, prolonged usage of broad-spectrum antibiotics, and having comorbidity. The microorganism may have resistance to most antimicrobial agents empirically prescribed for nosocomial Gram-negative infections. For this reason, when* C. indologenes* is isolated in normally sterile sites of body, antimicrobial susceptibility test results are important to assure appropriate antibiotic coverage. This case report demonstrated that TMP-SMX may be the only appropriate antimicrobial agent to treat bacteremia caused by this pathogen.

## Figures and Tables

**Table 1:** Demographic and clinical characteristics of *Chryseobacterium  indologenes* infections in pediatric patients.

Patient no.	Reference/year	Age/sex	Comorbidity	Medical devices present	Clinical syndrome	Antibiotic	Outcome
1	Hsueh et al., 1996 [2, 6]	1 mo/M	Burns	Ventilator	Ventilator-associated pneumonia	Ciprofloxacin, cefoxitin, amikacin	Died (ARDS)

2	Hsueh et al., 1996 [2, 6]	5 mo/F	Neuroblastoma, chemotherapy	Hickman catheter	Bacteremia	Not stated	Recovery without removal of catheter (after 3 days)

3	Hsueh et al., 1996 [2, 6]	1 mo/F	Hepatoblastoma, chemotherapy	Port-A-catheter	Bacteremia	Not stated	Recovery without removal of catheter (after 3 days)

4	Cascio et al., 2005 [13]	2 y/M	Type 1 diabetes mellitus	Peripheral catheter	Bacteremia	Ceftriaxone, 10 days	Recovery with removal of catheter (afebrile after 2 days)

5	Al-Tatari et al., 2007 [14]	13 y/M	Congenital hydrocephalus	Lumboperitoneal shunt	Lumboperitoneal shunt infection	TMP-SMX and rifampin (for 14 d after shunt removal)	Recovery 24 h after shunt removal

6	Bayraktar et al., 2007 [15]	5 mo/M	Down syndrome, operation for atrial septal defect and diaphragmatic hernia	Mechanical ventilation	Bacteremia	Vancomycin and ofloxacin	Died

7	Douvoyiannis et al., 2010 [16]	33 d/F	None	None	Bacteremia	Cefepime, 10 days	Recovery (afebrile after a day)

8	Ceylan et al., 2011 [17]	2 mo/M	Hydrocephaly	External shunt	Meningitis sepsis	Ampicillin sulbactam and levofloxacin	Died (cardiopulmonary arrest)

9	Calderón et al., 2011 [8]	20 d/M	Congenital heart disease	Mechanical ventilation	Ventilator-associated pneumonia	Piperacillin-tazobactam (14 days)	Recovery

10	Kodama et al., 2013 [18]	3 y/F	Acute myeloid leukemia unrelated cord blood stem cell transplantation	Central venous catheter	Catheter-related bloodstream infection	Ciprofloxacin and minocycline	Recovery with removal of catheter

11	Ozcan et al., 2013 [19]	6 mo/M	Congenital hydrocephalus, prematurity	Ventriculoperitoneal shunt	Meningitis	TMP-SMX and cefoperazone-sulbactam (14 days)	Recovery with removal of shunt

12	Hendaus et al., 2013 [20]	8 d/F	None	None	Meningitis	Cefepime (21 days)	Recovery (afebrile after 2 days)

13	Eshwara et al., 2014 [10]	6 d/F	Small for gestational age	None	Meningitis sepsis	TMP-SMX (2 weeks) and ciprofloxacin (6 weeks)	Recovery

14	Olbrich et al., 2014 [21]	11 mo/M	Holoprosencephaly, suboptimal hygienic conditions	Ventriculoperitoneal shunt	Meningitis	TMP-SMX and ceftazidime (21 days)	Recovery (afebrile in 24 h) with complete removal of the cerebrospinal shunt system

15	This study, 2014	3 mo/F	Congenital heart disease	Central venous catheter	Bacteremia	TMP-SMX (21 days)	Recovery (afebrile after 2 days)
